# In Silico Genomic Analysis of Chloroplast DNA in Vitis *Vinifera* L.: Identification of Key Regions for DNA Coding

**DOI:** 10.3390/genes16060686

**Published:** 2025-05-31

**Authors:** Francisca Peña, Luciano Univaso, Celián Román-Figueroa, Manuel Paneque

**Affiliations:** 1 Bionostra Chile Research Foundation, Almirante Lynch 1179, San Miguel 8920033, Santiago, Chile; fpena@bionostra.com (F.P.); lunivaso@bionostra.com (L.U.); croman@bionostra.com (C.R.-F.); 2 Department of Environmental Sciences and Natural Resources, Faculty of Agricultural Sciences, Universidad de Chile, Santa Rosa 11315, La Pintana 8820808, Santiago, Chile

**Keywords:** *Vitis vinifera*, chloroplast DNA, DNA barcoding, variety authentication, species conservation, phylogenetic analysis

## Abstract

Background/Objectives: The genus *Vitis* comprises approximately 70 species with high genetic diversity, among which *Vitis vinifera* is the most economically significant. Despite numerous studies on the genetic characterizations of *V. vinifera*, selecting optimal chloroplast DNA barcoding regions for intraspecific differentiation remains unresolved. Most studies have focused on nuclear markers (SSRs, SNPs) or widely used chloroplast loci (e.g., *matk*, *rbcl*), which have shown limited resolution at the subspecies level. In this study, the complete chloroplast genomes of 34 *V. vinifera* accessions from different varieties and hybrids (*vinifera*, *sylvestris*, *caucasica*, and *labrusca*) were analyzed to identify the key genomic regions for DNA barcoding. Methods: Using bioinformatics tools, we assessed the genome structure, nucleotide variability, microsatellites, codon usage bias, and phylogenetic relationships among the investigated varieties. Results: The chloroplast genomes displayed a quadripartite structure, with lengths ranging from 160,906 to 160,929 bp and a guanine–cytosine (GC) content of ~37.4%. Phylogenetic analysis revealed an unusual position for VV-5 vini and VVVL-3 lab, suggesting potential taxonomic misclassification or hybridization effects. A single locus showed low discrimination power, but the concatenation of five loci (*ccsA-trnN-GUU*, *rpl16*, *rpl2-rps19*, *rpoC2*, and *trnM-CAU*) exhibited significantly improved resolution (44.11% K2P), surpassing traditional markers. Conclusions: This study addresses the gap in the literature regarding the use of concatenated chloroplast loci for subspecies research; the results validate these markers across a broader range of *Vitis* accessions and integrate nuclear and mitochondrial data to achieve a more comprehensive understanding of the evolutionary history and genetic diversity of *V. vinifera*.

## 1. Introduction

The genus *Vitis* comprises approximately 70 species distributed in the temperate and subtropical regions of the Northern Hemisphere, with great genetic diversity and varied ecological adaptations [1]. Within this genus, *Vitis vinifera* L. is the most commercially and agriculturally relevant species, and it forms the basis of the global wine industry [2]. Its cultivation dates back thousands of years and has given rise to the various varieties used in producing wine, table grapes, and raisins [3]. The economic importance of *V. vinifera* lies not only in its role in food and beverage production but also in its cultural and historical value in multiple societies [4].

Two main subspecies are recognized within *V. vinifera*: *V. vinifera* subsp. *sylvestris*, considered the wild form, and *V. vinifera* subsp. *vinifera*, which has been domesticated and diversified into thousands of varieties throughout the history of viticulture [5]. These varieties, in an enological context, also known as strains, exhibit morphological, physiological, and genetic variations that influence their resistance to diseases, adaptation to different climates, and fruit quality for wine production [3].

The study and identification of these varieties are crucial for viticulture as they allow the preservation of genetic diversity and selection of genotypes with better agronomic characteristics and ensure the authenticity of products derived from the vine [6]. Historically, grapevine variety identification has been based on ampelography, a discipline that uses the morphological characteristics of leaves, bunches, and berries to distinguish varieties [7,8]. However, this method is limited because morphological traits can be influenced by environmental factors and the phenological state of the plant [1]. The development of molecular techniques has enabled the overcoming of these limitations by using genetic markers, such as microsatellites (also known as simple sequence repeats [SSRs]) and single-nucleotide polymorphisms (SNPs), which have proven to be highly effective tools for the identification and genetic characterization of grapevine varieties [3,9,10]. One of the most recent and promising approaches is the use of chloroplast DNA as a genetic marker, as the chloroplast genome is more conserved than the nuclear genome, allowing the precise identification of phylogenetic relationships and the detection of useful variations for the classification of subspecies and varieties [11].

DNA barcoding is a molecular technique that uses short and highly conserved DNA sequences to identify and differentiate species and subspecies. In plants, this method has been implemented mainly using chloroplast DNA regions such as *matK*, *rbcL*, and *trnH-psbA*, owing to their stability and ease of amplification [12]. In the case of *V. vinifera*, DNA barcoding has been used in various studies for variety authentication and traceability in the wine industry. For example, Pipia et al. [13] demonstrated that certain regions of chloroplast DNA can be distinguished with high precision between different grapevine varieties, facilitating cultivar identification and certification of origin in the production of high-quality wines. Previous studies have used chloroplast DNA barcoding to analyze genetic diversity in wild and domestic grape populations, providing valuable information on the evolution and adaptation of the species [5].

Despite advances in the genetic identification of *V. vinifera*, selecting optimal genomic regions for DNA barcoding remains challenging, especially because of the genetic variability within the species. Therefore, the present study aimed to analyze the genomic characteristics of *V. vinifera* chloroplast DNA to identify the most suitable regions for DNA barcoding, allowing for efficient differentiation between subspecies and varieties. Identification of these markers will contribute to the development of more precise molecular tools for the identification, certification, and conservation of genetic diversity in grapevines.

## 2. Materials and Methods

### 2.1. Genomic Data and Alignment

Complete chloroplast genomes were obtained from the National Center for Biotechnology Information (NCBI) database (https://www.ncbi.nlm.nih.gov/; accessed on 9 January 2024). Thirty-four *V. vinifera* genomes were included, belonging to the varieties/subspecies *vinifera* (vini), *caucasica* (cau), and *sylvestris* (syl), as well as hybrids of *vinifera* and *labrusca* (lab) (Table S1). For sequence alignment, MUMmer v3.1 [14] was used in the Linux command line with default parameters, using the chloroplast genome with GenBank code NC_007957.1 [15]. The chloroplast genome structure was determined using CPStools v2.0.2 [16] and IRscope web version [17] to visualize and analyze the boundaries of the large single copy (LSC), small single copy (SSC), and inverted repeat (IR) regions.

### 2.2. Variability and Genetic Coding Analysis

SSRs were identified in the chloroplast genomes of 34 *V. vinifera* varieties using MISA v2.1 [18], with the following criteria: a minimum of ten repeats for mononucleotides, five for dinucleotides, four for trinucleotides, and three each for tetranucleotides, pentanucleotides, and hexanucleotides. Long repeats were also identified using REPuter v1 [19], classifying direct (F), inverse (R), palindromic (P), and complementary (C) repeats with a minimum size of 20 bp and a Hamming distance of 3, as reported previously [20]. Relative synonymous codon usage (RSCU) analysis was performed on protein-coding genes, using the GFF3 files. Sharp and Li’s [21] criteria were applied, in which RSCU values < 1 indicated low codon usage, RSCU = 1 indicated unbiased usage, and RSCU > 1 indicated high usage [22]. Additionally, RSCU values < 0.6 represented very low usage, whereas values > 1.6 indicated very high usage [23]. Mutations in genes and intergenes, including SNPs and insertions–deletions (indels), were analyzed using MUMmer v3.1 and Geneious v9.1.8 [24]. For Geneious, manual analysis of SNPs and indels was performed for each gene and intergene.

### 2.3. Phylogenetic Analysis and DNA Barcoding

To evaluate the discriminatory capacity of genes and intergenes as potential candidates for DNA barcoding, divergence analysis was conducted using the two-parameter Kimura distance (K2P) [25]. Since the resolution percentages obtained were less than 50%, gene and intergene concatenations were performed to enhance their values. Phylogenetic analysis was carried out using RAxML v8.2.12 [26], employing the GTR model and 5000 bootstrap replicates with the fast bootstrapping method [26]. The resulting phylogenetic trees were visualized in R using the ggtree library [27].

## 3. Results

### 3.1. General Characteristics of Chloroplast DNA

The chloroplast genome of *V. vinifera* displayed a typical quadripartite circular structure, with a total length ranging from 160,906 to 160,929 bp, corresponding to VVSC-4_cau and VVSS-19_syl, respectively. The reference genome utilized was that of VV-1_vini, as reported by NCBI, with a length of 160,928 bp, and shared by 21 other genomes. These genomes consisted of an LSC region 89,135 or 89,194 bp long and an SSC region 19,071 or 19,073 bp long, separated by a pair of IRa and IRb regions 26,353 or 26,356 bp in length, respectively (Table S1).

In most genomes, the LSC region initiated at base 1 and concluded at base 89,140 ± 10 bp, although two specific cases (VV-5_vini and VVVL-3_lab) exhibited displaced starts and ends: VV-5_vini began at base 47,136 and ended at 136,282, while VVVL-3_lab started at base 71,781 and ended at 160,928. Additionally, 28 genomes had an LSC region that terminated between bases 160,920 and 160,928 (Table S2).

The guanine–cytosine content (%GC) varied slightly between regions; the entire chloroplast region had between 37.38% and 37.40% GC, whereas the IRa and IRb regions had between 43.03% and 42.96% GC. In contrast, the SSC region presented the lowest GC content, between 31.69% and 31.66% (Tables S1 and S3).

The total number of genes ranged from 164 to 180, with the number of protein-coding genes ranging from 121 to 134. The number of genes encoding tRNA ranged from 35 to 38, while the number of genes encoding rRNA ranged from six to eight (Table S4). The number of intergenic regions identified ranged from 104 to 119 per variety (Table S4).

The identified protein-coding genes included six genes related to photosystem I, fifteen genes related to photosystem II, six genes associated with ATP synthesis, eleven genes related to large ribosomal proteins, and twelve genes related to small ribosomal proteins. Some genes, such as *rps7*, *rpl2*, *rpl23*, and *rps12*, were duplicated (Table S5).

### 3.2. RSCU Profile Analysis

**Figure 1:**
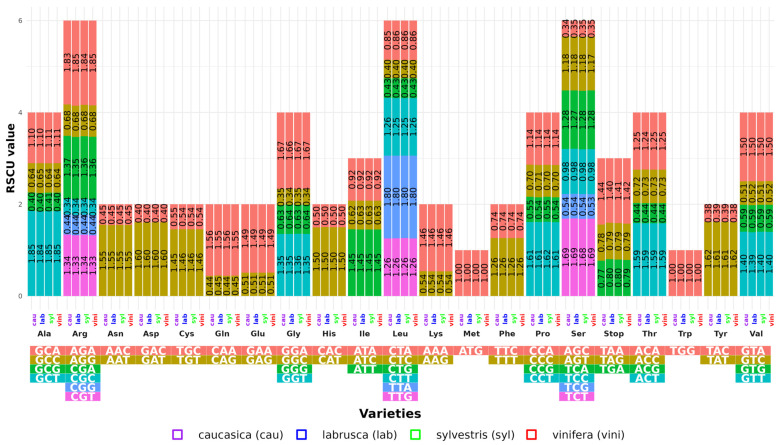
Relative synonymous codon usage for amino acids in the protein-coding regions of the chloroplast genomes of the 34 *Vitis vinifera* varieties investigated in this study.

The RSCU for the protein-coding genes in the cp genome of each variety was also calculated. These genes encode 21 amino acids with 64 codons (Figure 1). The number of codons varied from 26,006 in VVVC-3_cau to 26,835 in VV-5_vini (Table S6). Among the amino acids, leucine (Leu) was the most abundant, encoding an average of 2744 codons (10.09%). VVVL-3_lab had the highest number of Leu codons, while VV-2_vini and VVVL-1_lab had the lowest counts. In contrast, cysteine (Cys) was the least abundant amino acid, encoded by an average of 342 codons (1.18%) (Table S7).

Although the varieties exhibited similar RSCU patterns, differences were observed in the termination codons, with proportions of TAA (1.14%) being higher than those of TGA and TAG (0.79%). Among the four varieties, 32 codons had RSCU values > 1, while the other 32 showed values < 1. The GCT codon had the highest RSCU value (1.85 in vini and syl), followed by AGA (1.84 in lab and 1.83 in cau). In contrast, the CGC and GGC codons had the lowest values (0.34).

Methionine (Met) and tryptophan (Trp) exhibited unbiased use (RSCU = 1). Particularly, codons ending in A or T (such as GAT, CCT, TAT, and GGA) showed a high bias (RSCU > 1.6), while 20 codons ending in C or G had very low biases (RSCU < 0.6).

### 3.3. Variability in the LSC, SSC, and IR Regions

A comparison of chloroplast genomes among *V. vinifera* varieties revealed that the regions maintained a similar organization in all analyzed genomes, indicating a high degree of structural conservation. Figure S1 illustrates these differences, along with the precise measurements of the lengths of each region, showing minor changes that do not alter the overall structure. The junctions defining the boundaries between the LSC, IR, and SSC regions were also examined, demonstrating a highly conserved structure among laboratory varieties (VVVL-1, VVVL-2, and VVVL-3), where the junctions around the *rps19*, *ndhF*, *ycf1*, and *psbA* genes remained consistent. In contrast, the syl varieties (VVSS-1_syl to VVSS-20_syl) displayed greater variability in junctions, especially near ycf1 and ndhF, compared to the other varieties.

In the vini varieties (VV-1_vini to VV-5_vini), the junctions were more uniformly aligned with those in the laboratory, although small variations were observed in trnH-GUG and rpl22. Finally, the cau variety (VVSC-1_cau to VVSC-6_cau) presented mixed patterns, with some junctions coinciding with those of the lab and others with displacements similar to those observed in the syl.

### 3.4. Simple Sequence Repeats (SSRs) and Long Sequence Repeats

#### 3.4.1. SSRs

**Figure 2:**
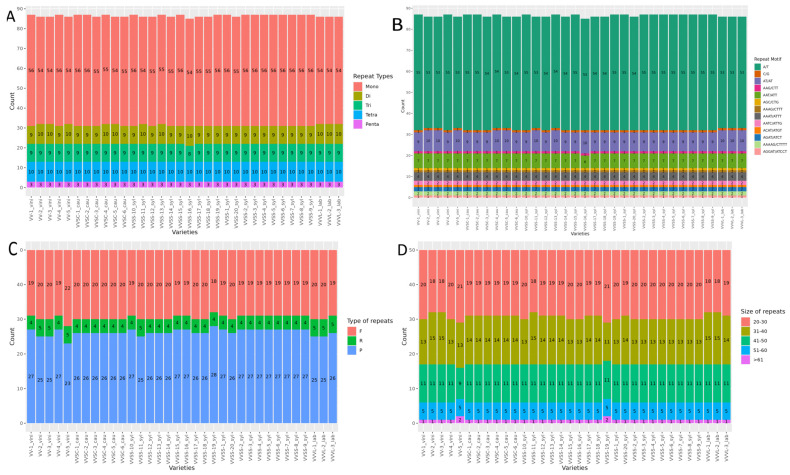
Simple sequence repeats (SSRs) and long repeats in the chloroplast genomes. *X*-axis: varieties. *Y*-axis: SSR count. (**A**) Repeat types of SSRs. (**B**) Frequency of SSRs in different repeat class types. (**C**) Number of long-repeat sequences (F: forward repeats; R: reverse repeats; P: palindromic repeats). (**D**) Comparison of long repeats based on size.

Five types of SSR motifs were identified: mononucleotides (Mono), dinucleotides (Di), trinucleotides (Tri), tetranucleotides (Tetra), and pentanucleotides (Penta). Mono motifs were the most abundant among all varieties studied, with values ranging between 54 and 56 repeats. In contrast, Penta motifs were the least frequent, with a constant distribution of three repeats across all varieties. Di, Tri, and Tetra motifs showed a more balanced distribution, with counts varying between nine and ten repeats (Figure 2A).

The most frequent motifs were mononucleotides A/T, with counts ranging between 53 and 55 repeats, depending on the variety (Figure 2B). The dinucleotide AT/AT presented a lower frequency (between nine and ten repeats) among the investigated varieties. Among the trinucleotides, the AAT/ATT motif was identified at a frequency of seven repetitions in most varieties. In tetranucleotides, motifs such as AAAT/ATTT, AATC/ATTG, and AGAT/ATCT appeared in smaller numbers, at four, two, and two repeats, respectively. Pentanucleotide motifs, such as AAAAG/CTTTT and AGGAT/ATCCT, had even lower frequencies, with only two and only one repeat, respectively, in all varieties.

#### 3.4.2. Long Repeats

Forward repeats were the most common among all the varieties (18–22), followed by palindromic repeats (23–28). Reverse repeats (between four and five) were less frequently observed, whereas complementary repeats were not recorded. Significant differences in the total number of long repeats were identified among the varieties (Figure 2C). Analyses of long repeats in the chloroplast genomes of the *V. vinifera* varieties showed a similar distribution pattern in five length categories: 20–30 bp, 31–40 bp, 41–50 bp, 51–60 bp, and >61 bp (Figure 2D). The shortest length (20–30 bp) was the most frequent in all varieties, ranging from 18–21 repeats. Repeats in the range of 31–40 bp (between 13 and 15) followed this, whereas those 41–50 bp long presented a constant value of 11 repeats in most of the analyzed genomes, except VV-5_vini. Repeats of 51–60 bp were uniform in all varieties, with five occurrences, whereas those larger than 61 bp were the least common, with one or two occurrences depending on the variety.

### 3.5. SNPs and Indels in the Genome

**Figure 3:**
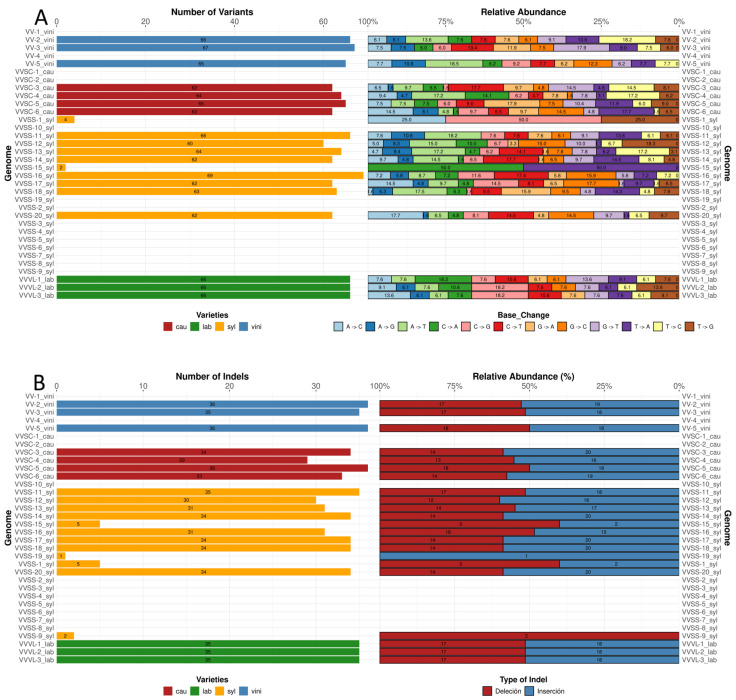
Genetic variation in *Vitis vinifera* chloroplast genomes. (**A**) Number of single-nucleotide polymorphism (SNP) variants and relative abundance of base changes per genome. The graph on the left shows the total number of SNP variants identified per genome, whereas the graph on the right indicates the relative abundance (%) of the different types of base changes (A → G, A → T, C → A, etc.). (**B**) Number of indels and relative abundance of insertions and deletions per genome. The graph on the left shows the total number of insertions and deletions identified per genome, and the graph on the right shows the relative distribution (%) of insertions and deletions. In both cases, colors represent the varieties (vini, cau, syl, and lab), and genomes are labeled on the *y*-axis.

The total number of SNPs per genome ranged from 62 to 69 (Figure 3A), with the syl variety exhibiting the highest variability and the vini and lab exhibiting the lowest. Notably, there are varieties (14) that did not present SNPs, whereas two varieties severally presented four and two SNPs. Regarding the relative abundance of base changes, transition types (C → T and A → G) were the most frequent among all varieties, representing the largest proportion of the identified SNPs. Transversal changes, such as T → A and G → T, were less common, although their contribution varied slightly between varieties. In particular, C → T changes reached higher proportions in the genomes of the cau variety, whereas a more balanced distribution between A → G and C → T changes was observed in the syl variety. The lab variety showed a pattern similar to vini, with a prominent prevalence of A → G transitions (Figure 3A).

The number of indels per genome varied between 29 and 36; in some cases, it was less than five. The vini variety contained 35 or 36 indels in all the analyzed genomes. In contrast, cau showed a higher variability, with a range of 29–34 indels. Syl presented values close to the average, with most genomes showing 31–35 indels. In contrast, lab varieties had an intermediate number of indels, at 35. Regarding the relative distribution of indel types, deletions and insertions were present in equal proportions. Vini presented between 17 and 18 deletions and between 18 and 19 insertions. The cau population exhibited between 13 and 18 deletions and 16 and 20 insertions. The syl subspecies showed the greatest variation, with deletions ranging between 3 and 18 and insertions ranging between 1 and 20. Similar values were observed in lab varieties: 17 deletions and 18 insertions (Figure 3B).

SNPs and indels were distributed throughout the genome (Figure S2), with a higher proportion in the LSC region relative to the IR and SSC regions. In addition to the general patterns of SNP and indel distributions, particular differences were observed among *V. vinifera* varieties. For example, the vini and syl varieties had a higher density of mutations in the LSC region. In contrast, the cau and lab subspecies showed a more homogeneous distribution of mutations, with a lower proportion in the IR regions. However, individual genomes within each subspecies also showed differences in the total numbers of SNPs and indels. The SSC region presented a clear pattern of SNP and indel distributions across the analyzed genomes. Although the mutations were not uniform, some genomes exhibited specific clusters in this region.

### 3.6. Evolutionary Relationships Between V. vinifera Chloroplast Genomes

**Figure 4:**
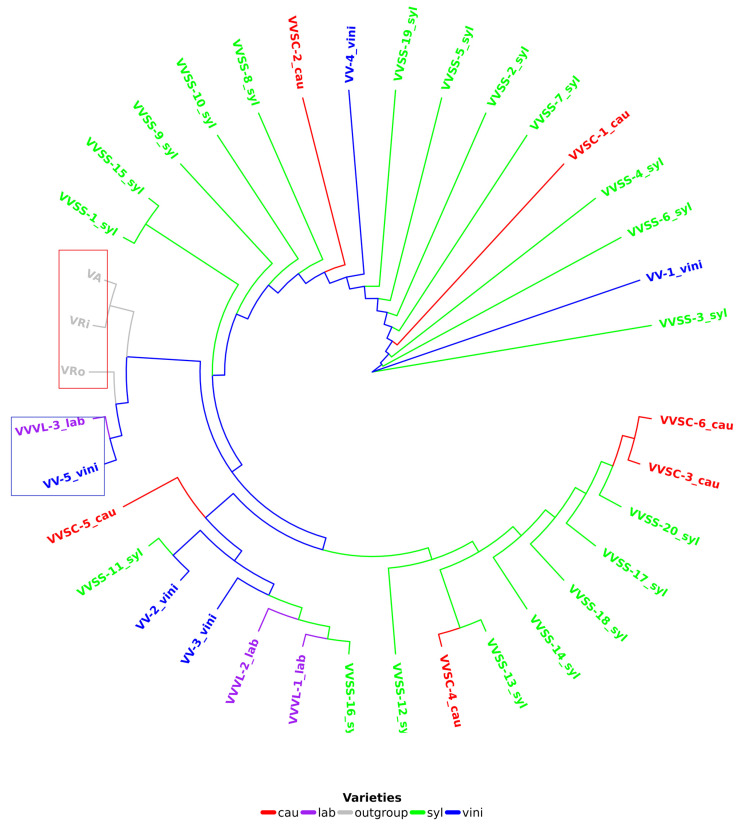
Reference phylogenetic tree of *Vitis vinifera* varieties based on 34 chloroplast genomes of *V. vinifera* and three chloroplast genomes of species of the genus *Vitis* (*V. riparia*, *V. aestivalis*, and *V. rotundifolia*) used for rooting. Different colors represent the various varieties of *V. vinifera.*

A reference phylogenetic tree (Figure 4) was constructed for all grapevine cp genomes (34, Table S1), as well as three Vitis species (*V. riparia* Michx, *V. aestivalis* Michx, and *V. rotundifolia* Michx) considered as external groups to root the tree and discriminate between grapevine varieties/cultivars. In this tree, it was observed that samples VV-5 vini and VVVL-3 lab were positioned before the external groups, indicating a common ancestor between these samples and the rooting species (Figure 4). The external groups were different species within the same genus; therefore, they were expected to be found further away in the tree. VVVL-3 lab is a hybrid of V. vinifera and V. labrusca.

### 3.7. Divergence Analysis Based on K2P and Phylogenetic Analysis of Loci (Genes and Intergenes)

According to the K2P value, the resolution of a locus revealed a low discriminatory capacity for genes (between 0.0% and 8.82%), with a slightly higher capacity for intergenes (between 0.0% and 17.65%; Table S8). The phylogenetic trees constructed for the ndhD and trnR-ACG genes (independently) showed one and three differentiated clades (Figure S3), respectively. It was not feasible to observe groupings between grapevine varieties (vini, syl, and cau) or between hybrids (lab). Furthermore, the VV-5 vini and VVVL-3 lab sequences, which were found together in the reference tree and far from the rest of the grapevine samples (Figure 4), did not group in either tree and were found in different clades (Figure S3). In contrast, when constructing the phylogenetic tree with the petD-ycf2 intergene, a higher number of clades (five; Figure S4) was observed compared to those constructed with the *ndhD* and *trnR-ACG* genes (one and four, respectively; Figure S3). It was also observed that the VV-5 vini and VVVL-3 lab sequences were grouped within the same clade as the other grapevine sequences. However, the clades showed high diversity in their conformation; therefore, it was possible to find all grapevine subspecies mixed in the clades (Figure S4).

**Figure 5:**
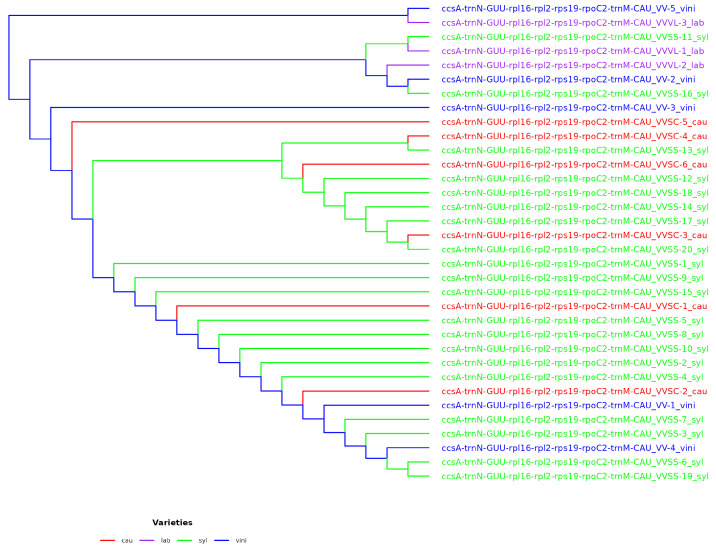
Phylogenetic tree for *Vitis vinifera* varieties, based on the concatenation of five loci for *ccsA-trnN-GUU_rpl16_rpl2-rps19_rpoC2_trnMCAU*, through the neighbor-joining (NJ) method, for the 34 chloroplast genomes obtained from GenBank. Parameters: 5000 initial bootstrap tests using the GTR+I+G substitution model.

The use of intergenes showed a greater resolution capacity in the phylogenetic trees, but was not sufficient to resolve the phylogeny. The resolution capacity when concatenating genes, intergenes, and their combination increased the K2P value, which reached 20.59% when combining two genes (4371 combinations), 35.29% when combining gene_intergene (5585 combinations), and 32.35% when combining two intergenes (1622 combinations). The concatenation of two loci (gene_gene, gene_intergene, and intergene_intergene) was not resolved; therefore, the concatenation of three or more loci was evaluated considering various combinations. The best combination of five loci (ccsA-trnN-GUU_rpl16_rpl2-rps19_rpoC2_trnMCAU), which showed the same resolving capacity according to the K2P value (44.11%), was also the one that placed the varieties VV-5 vini and VVVL-3 lab in the same clade and ancestored the rest of the phylogenetic tree (Figure 5). However, the configuration of the phylogenetic tree elaborated with five loci showed similarities in its conformation with the reference tree (Figure 4) in terms of the quantity and structure of the clades, although not necessarily the same distribution of subspecies for each clade.

## 4. Discussion

This study presented a detailed analysis of the chloroplast genomes of various *V. vinifera* varieties, focusing on the identification of key regions involved in their molecular differentiation. The results revealed a high degree of genomic conservation. However, subtle differences were observed in the structures of the LSC, SSC, and IR regions and in the patterns of variability of SNPs and indels. The findings are subsequently discussed in comparison with those of previous studies, and in their relevance to the taxonomy and evolution of *V. vinifera*.

### 4.1. Data Used

Some *V. vinifera* accessions (NC_007957.1, MN561034.1, MW592542.1, DQ424856.1, and OR500062.1) lacked detailed information about their subspecies in the GenBank records. Correct taxonomic classification is crucial for comparative analyses; therefore, these accessions were grouped within the *V. vinifera* vini group (referring to the *vinifera* subspecies) based on a parsimony criterion, considering the dominance of this subspecies in previous chloroplast genome studies [28,29]. This methodological choice enabled our analyses to include all relevant accessions without exclusion. It is important to note that the observed differences between groups may signify variations between subspecies and intraspecific differences within *V. vinifera*. Previous studies have highlighted significant genetic variability within this species, even within the same subspecies or variety [5,30]. Hence, the findings of this analysis should be interpreted cautiously and re-evaluated in future studies incorporating a more detailed characterization of these accessions.

Furthermore, the utilization of up-to-date bioinformatics tools and databases could enhance the classification of these accessions. Recent research has demonstrated that the integration of phylogenetic analysis with SNP-based identification methods can effectively distinguish subspecies and varieties of *V. vinifera* [31,32]. Future studies should employ comprehensive comparative approaches to confirm or adjust the taxonomic assignments of these accessions.

### 4.2. General Features of the Chloroplast Genome

The analyzed *V. vinifera* chloroplast genomes exhibited quadripartite organization, with lengths ranging from 160,906 to 160,929 bp, consistent with those reported for other *Vitis* species [20]. The high structural conservation of the IR regions aligns with studies on angiosperms and *V. vinifera* landraces, suggesting the roles of these regions in genomic stability [11,33]. However, slight variations in the lengths of the IR and SSC regions among the studied subspecies, particularly in VVSS-2 SYL and VVSS-6 SYL, indicate that genetic events may be linked to specific evolutionary adaptations. These differences underscore the potential of these regions for molecular classification, as mutations in conserved regions could indicate divergent evolutionary paths [34,35,36].

Notably, the IRa and IRb regions exhibited higher levels of conservation in their GC content, while the more variable SSC region could be pivotal in distinguishing between subspecies. The LSC region also displayed intriguing variability patterns that could complement the differences observed in the SSC region, providing additional information to differentiate between varieties. These observations lay the groundwork for exploring instances of genomic variability in specific segments and linking them to more detailed SNP and indel analyses.

The slight variations in the IR/LSC and IR/SSC junctions in some varieties might be associated with genomic rearrangement events or adaptations to diverse ecological environments [36]. However, it is worth mentioning that, for the most part, the total number of base pairs in the LSC region did not significantly differ from those observed in other varieties, with an average length of 89,140 ± 10 bp. The variations in the start and end of this region in the genomes of VV-5_vini and VVVL-3_lab (starting at 47,136 and 71,781 bp and ending at 136,282 and 160,928 bp, respectively) did not substantially impact the total size of the region, suggesting that these variations did not affect the genome’s functionality. This variability in the LSC region may indicate local evolutionary processes that do not entail major changes in the overall genome structure but could reflect specific adaptations to varying environmental or ecological conditions.

### 4.3. Codon Usage Bias

The codon usage index (RSCU) revealed interesting patterns of synonymous selection, with the most optimal synonymous codons ending in A or U in higher plants [37]. Analysis of the *Vitis* genome showed that Leu is one of the most frequent amino acids, whereas Cys is underrepresented. This distribution is not random but is influenced by the nucleotide composition of the DNA, particularly the GC content. Previous studies have shown that organisms with high GC content in their genomes tend to prefer codons rich in these nucleotides, which directly affect the frequency of certain amino acids in proteins. Because codons encoding Leu contain more G and C in their synonymous positions, their use is favored in genomes with high GC content. In contrast, Cys, which is encoded by codons with lower GC proportions, was found in lower quantities in these genomes. These results are consistent with previous studies linking GC composition to codon usage bias and amino acid distribution in proteins [22,38,39]. Furthermore, high RSCU values for codons ending in A or U(T) (between 1.84 and 1.26) may be related to subspecies-specific evolutionary features [39]. Investigating codon usage bias is essential for understanding codon usage patterns in closely related species [40]. This knowledge is valuable for exploring genetic evolution, deciphering gene expression features, and providing guidance for breeding [35,41].

Elucidating the patterns of codon usage bias also complemented the analysis of variability in the LSC and SSC regions; the density of mutations in coding genes could be influenced by this bias. This finding underlines the importance of considering both genomic structure and codon usage when identifying key regions for DNA barcoding.

Although codon usage bias is not a direct criterion for the taxonomic classification of *V. vinifera* subspecies, its analysis was included as part of a comprehensive characterization of the chloroplastidial genomes analyzed. This approach aims to provide a comprehensive view of the composition and evolutionary behavior of the coding genes to support future research on selection pressure, translation efficiency, or gene expression. While this information was not used as a criterion for loci selection in DNA barcoding analyses, it represents a useful informative complement within the overall comparative context of the study.

### 4.4. Simple and Long Repeats

Analysis of SSRs in the chloroplast genomes of *V. vinifera* showed that their distribution was not random, with a marked predominance of A/T mononucleotides (55–53 between varieties), which is consistent with previous studies on angiosperms [42,43]. This overabundance of A/T in chloroplastidial SSRs can be explained by the lower stability of A-T base pairs compared to that of G-C pairs, which facilitates the expansion and contraction of these repeats throughout evolution [44]. Because A/T-rich DNA regions are more prone to mutations owing to their lower structural stability, the high frequency of A/T mononucleotides in the chloroplast may reflect a higher rate of recombination or replication errors in these repetitive sequences [45].

However, differences in the number and type of repeats were observed among varieties, suggesting that these elements may be subject to specific selective pressures. In particular, the frequency of palindromic repeats (28–23) and the absence of complementary repeats in certain varieties may be associated with differences in the structural stability of the chloroplast genome and its gene regulation capacity. Palindromic repeats are regions prone to forming secondary DNA structures that can affect gene replication and expression [46]. The variability in the number of repeats among *V. vinifera* varieties could reflect adaptations to different ecological environments, in which certain repeat patterns could confer selective advantages in the stability of the chloroplast genome or transcription efficiency. Furthermore, the absence of complementary repeats in some varieties suggests possible elimination or differential mutations in these regions, possibly related to genetic drift or purifying selection in isolated populations. Previous studies have shown that complementary repeats are less frequent in chloroplastidial genomes owing to their lower structural stability and greater susceptibility to unequal homologous recombination, which may explain their absence in certain varieties [36].

Another key finding was the correlation between regions of high SSR density and areas of high genomic variability identified in the SNP and indel analyses. This suggests SSRs act as mutation hotspots and contribute to intraspecific genetic differentiation. It has been proposed that regions with high repeat densities serve as foci for genomic instability, facilitating the generation of new genetic variants that could influence the local adaptation of *V. vinifera* varieties [47].

In the context of chloroplast evolution in *Vitis*, the results of our study reinforce the idea that the distribution of SSRs is not only a reflection of the conserved genomic architecture but can also be modulated by specific evolutionary pressures. Variability in the composition and abundance of repeats among varieties can provide valuable information on the processes of population differentiation and natural selection that affect *V. vinifera*.

Long repeats, classified into four types ranging in size from 20 to 61 bp, also exhibited variety-specific patterns. Collectively, our results reinforce the potential of SSRs and long repeats as tools for genetic characterization and intraspecific differentiation in *V. vinifera*, supporting their use in phylogenetic and genetic diversity studies [48,49].

### 4.5. Variability Patterns: SNPs and Indels

The distribution pattern of SNPs and indels in the chloroplast genome reaffirmed the more highly variable nature of the LSC region (Figure S2) compared to the IR and SSC regions. The dominant transitions (C → T and A → G, Figure 3) in all varieties reflect common evolutionary trends, except in cases where these mutations are absent and/or occur in low proportion (VVSS-1_syl and VVSS-15_syl). Furthermore, the less frequent transversions (A → T, A → C, G → T, C → G) highlight variety-specific differences, such as those observed in cau and syl. C → T and A → G transitions are predominant, suggesting conserved evolutionary dynamics in *Vitis* [25]. Notably, samples from the VV-5 vini and VVVL-3 labs present a significantly higher number of mutations in the IR region, which could indicate evolutionary divergence or errors in taxonomic classification. This phenomenon has been reported in other species, in which hybridization has influenced the organization of the chloroplast genome [50].

### 4.6. Gene and Intergene Resolution Capacity

Subspecies identification within *V. vinifera* represents a considerable challenge in DNA barcoding, as most previous studies have focused on the interspecific or intergeneric levels, leaving a gap in resolution at the intraspecific level [51]. Traditionally, genes such as *matK* and *rbcL*, and intergenes such as *trnH-psbA* and *trnL-trnF*, have been widely used for plant species identification [12,52]. However, in the present study, these markers showed low resolution when applied to *V. vinifera* varieties (Table S8), reinforcing the need to explore new genomic regions with greater discriminatory power.

The current analysis identified alternative gene and intergene combinations with better performance in subspecies differentiation. In particular, evaluating *rpoC*, *trnM-CAU*, and *rpl16* (with a resolution of 2.94% each when evaluated independently; all of these are present in the LSC region of the chloroplast), and the intergenes *ccsA-trnN-GUU* and *rpl2-rps19* (with a resolution of 11.76% each when evaluated separately; they are present in the SSC region and at the end and beginning of the IRa and LSC regions, respectively) proved to be more effective than using traditional markers. However, it is important to highlight that although *ccsA-trnN-GUU* has already been reported as a divergent intergenic region [53], *rpl2-rps19* has not been previously considered a barcode marker in subspecies identification studies, highlighting the originality of the current findings. In contrast, *rpoC* and *rpl16* are highly effective in phylogenetic studies of other plant species [54,55,56]. However, no previous studies have reported using *trnM-CAU* as a viable candidate for DNA barcoding, suggesting that its inclusion in future studies could significantly expand the tools available for intraspecific identification of *V. vinifera*.

A key aspect of this study was the evaluation of multiple loci concatenation as a strategy used to improve phylogenetic resolution. The combination of *ccsA-trnN-GUU*, *rpl16*, *rpl2-rps19*, *rpoC2*, and *trnM-CAU* achieved 44.11% resolution, representing a considerable advancement compared to a single locus. This approach allowed better differentiation between subspecies and reflected phylogenetic patterns consistent with reference trees, reinforcing the effectiveness of concatenation strategies in the improvement of DNA barcoding accuracy [57].

In addition to comparisons with conventional barcode studies, comparing these results with those of other methodologies used to identify varieties and subspecies of *V. vinifera* is essential. For example, the Institute of Agricultural Research has used techniques based on molecular markers such as SSRs and SNPs in varietal identification studies [58]. Although these techniques have proven highly effective in discriminating between cultivated varieties, their applicability to differentiation at the subspecies level remains limited because of the high genomic conservation within *V. vinifera*.

Nuclear SNPs are another widely used strategy for the genetic characterization of *Vitis*; however, they require more data and more complex computational analyses. In contrast, DNA barcoding based on chloroplast genomes offers advantages in terms of a lower recombination rate and maternal inheritance, making it a complementary tool for intraspecific identification [50]. This study highlights that the success of barcoding in *V. vinifera* is highly dependent on the appropriate selection of loci, underlining the importance of approaches such as the concatenation of multiple genomic regions. These results reinforce the idea that exploring new loci within the chloroplast genome can significantly improve intraspecific resolution, complementing traditional techniques based on nuclear DNA. Furthermore, discovering novel loci with greater discriminatory power opens the door for future research aiming to validate these markers for varietal identification and genetic conservation within the *Vitis* genus.

### 4.7. Phylogenetic Position of VV-5 Vini and VVVL-3 Lab

A key finding of this study was the atypical phylogenetic placement of the accessions VV-5 vini and VVVL-3 lab, which appeared before the outgroups in the *V. vinifera* phylogenetic tree. This unusual position suggests that these accessions might not belong to *V. vinifera* or might represent lineages with a distinct evolutionary history. In particular, VVVL-3 lab, a hybrid of *V. vinifera* and *V. labrusca*, seems to explain this anomaly because hybrids often present complex genetic features that do not fully align with their parental species [59,60]. This phenomenon has been previously documented in hybridization studies within the genus *Vitis* and highlights the importance of considering species interactions in interpreting phylogenetic relationships [1].

However, VV-5 vini presents a more intriguing challenge, as its position in the phylogenetic tree requires further analysis. Several hypotheses can explain this finding. First, there could have been a mistake in classifying sequences in public databases, which could have influenced the erroneous assignment of its place in the phylogenetic tree [49]. This underlines the importance of reviewing and validating genomic sequences using multiple tools and databases to avoid taxonomic errors. Another plausible hypothesis is that VV-5 represents a previously undescribed lineage of the *V. vinifera* complex. The observation of an early divergence in its phylogenetic tree could indicate the existence of an isolated population with unique evolutionary characteristics, suggesting that the genetic diversity within *Vitis* might be underestimated [61]. These findings reinforce the need to further explore *V. vinifera* populations in diverse geographical regions, as there may be unidentified lineages that contribute to a better understanding of the evolutionary history of this species. Furthermore, the possibility that VV-5 vini resulted from a recent evolutionary process cannot be ruled out. The ongoing speciation phenomenon, driven by ecological, geographic, or reproductive factors, can lead to the early genetic differentiation of certain lineages of *V. vinifera* [3]. This hypothesis opens the door for future research exploring how environmental and genetic factors influence the evolution of *Vitis*.

Moreover, the analysis of VVVL-3 lab highlights the importance of studying the hybrids between *V. vinifera* and other *Vitis* species, such as *V. labrusca*. These hybrids may play important roles in the genetic diversification and adaptation of species to different environmental conditions [3]. Furthermore, understanding the genetic interactions between these species will help clarify how hybrids can influence genetic variability within the *Vitis* genus.

Furthermore, previous studies on the genetic diversity of *V. vinifera* employed nuclear SSR and SNP markers [3,10]. However, chloroplast DNA offers advantages for phylogenetic reconstruction because of its low recombination rate and maternal inheritance [11]. This study adds to the recent research that has proposed new combinations of loci to improve genetic resolution in species with high genomic conservation, such as that reported by Waswa et al. [53] for other botanical families.

### 4.8. Limitations of the Study

An important limitation of the present study is that the taxonomic assignments of the analyzed accessions were taken directly from publicly available databases (GenBank) at the time of downloading the chloroplastidial genomes. In some cases, such as VV-5_vini and VVVL-3_lab, this classification could be inaccurate or incomplete, which would explain their atypical phylogenetic positions. While the behavior of VVVL-3_lab may be associated with its known hybrid origin (*V. vinifera* × *V. labrusca*), the basal position of VV-5_vini suggests an unresolved evolutionary history, one possibly related to misclassification, a divergent lineage, or a lack of previous characterization. Because only a minority of these accessions have available nuclear or mitochondrial sequences, it was not possible to integrate multiple levels of genomic analysis. Therefore, caution is advised when interpreting extreme or unexpected groupings, and the need for future research that includes accessions with expert-confirmed taxonomic identification, as well as the use of multilocus information to strengthen intraspecific phylogenetic analyses in Vitis vinifera, is recognized.

Furthermore, the possibility that accessions such as VV-5_vini and VVVL-3_lab are related to species outside the *V. vinifera* complex, such as *Vitis rotundifolia* or other species of the *Muscadinia* subgenus, cannot be ruled out, especially if interspecific hybridization or chloroplastidial capture has occurred. This hypothesis could explain their basal phylogenetic position close to the outgroups and highlights the need to revise the original classification and perform integrative genomic studies in future work.

## 5. Conclusions

In conclusion, this study provided an in-depth understanding of chloroplast genomic structure and variability in different *V. vinifera* varieties. Identification of key genomic regions and analysis of elements such as SNPs, indels, simple and long repeats, and codon usage revealed substantial differences between subspecies, highlighting the potential of these techniques as DNA barcoding tools. Although high structural conservation was confirmed in the analyzed genomes, differences in the SSC and LSC regions and the phylogenetic positions of certain accessions suggest possible evolutionary divergence or errors in taxonomic classification. This variability highlights the potential of genomic differences as a molecular marker for differentiating between closely related varieties, particularly in taxa with high genetic diversity, such as *V. vinifera*. Furthermore, the combination of multiple loci, such as *ccsA-trnN-GUU_rpl16_rpl2-rps19_rpoC2_trnM-CAU*, significantly increased the phylogenetic resolution to 44.11%, overcoming the limitations of using individual loci. The identification of novel loci combinations with improved resolution capabilities, such as *ccsA-trnN-GUU_rpl16_rpl2-rps19_rpoC2_trnM-CAU*, underscores the importance of expanding beyond the traditionally used barcoding loci, such as *matK* and *rbcL*.

Collectively, the findings in this study open new avenues for phylogenetic studies and species authentication applications. These results underline the importance of the integration of advanced concatenation strategies and genomic analyses to address molecular classification and species authentication challenges. This framework can be extended to other biological systems with high genetic diversity, opening new possibilities for molecular biology and evolution research. Moreover, these findings may be particularly useful in the wine industry for ensuring cultivar authenticity and tracing product origins. Furthermore, using the specific loci identified in this study could facilitate genetic conservation studies, helping preserve the diversity of *V. vinifera* subspecies in the face of environmental and economic threats. Future studies should focus on validating these findings in a broader range of grapevine cultivars and integrating nuclear and mitochondrial genome data to build a more complete understanding of the evolutionary history and genetic architecture of *V. vinifera*.

Finally, this study highlights the importance of moving toward integrative approaches that combine chloroplastidial, nuclear, and mitochondrial genomic information, along with accessions with verified taxonomic classification, to improve the resolution of evolutionary relationships within *V. vinifera*. These efforts will allow not only better delimitation between subspecies and varieties, but also greater confidence in the phylogenetic interpretation of lineages with unusual or divergent characteristics.

## Data Availability

The original contributions presented in this study are included in the article/Supplementary Materials. Further inquiries can be directed to the corresponding author.

## Supplementary Materials

The following supporting information can be downloaded at: https://www.mdpi.com/article/10.3390/genes16060686/s1. Figure S1. Comparison of the boundaries of the LSC, SSC, and IR regions among the 34 *Vitis vinífera* varieties investigated in this study. Figure S2. Distribution of single nucleotide polymorphisms and indels in the *Vitis vinifera* genome: (a) 32 genomes of *Vitis vinifera* varieties; (b) VV-5_vini and VVVL-3_lab genomes. Figure S3. Phylogenetic tree for *Vitis vinifera* varieties, based on the ndhD gene (a) and trnR-ACG (b), using the neighbor-joining (NJ) method for the 34 chloroplast genomes obtained from GenBank. Parameters: 5000 initial bootstrap tests using the GTR+I+G substitution model. Figure S4. Phylogenetic tree for *Vitis vinifera* varieties, based on the petD-ycf2 intergene, using the neighbor-joining (NJ) method for the 34 chloroplast genomes obtained from GenBank. Parameters: 5000 bootstrap tests using the GTR+I+G substitution model. Table S1. Summary of the complete chloroplast genome characteristics of *Vitis vinífera* varieties. Table S2. Start and end for each of the four regions comprising the chloroplast genome for each of the 34 varieties. Table S3. Percentage of guanine and cytosine (%GC) for each of the four regions comprising the chloroplast genome for each of the 34 varieties. Table S4. K2P values (%) for gene and intergene resolution used to discriminate between subspecies and/or varieties of *Vitis vinifera*. Table S5. Summary of the genes, tRNA, and rRNA for the Vitis vinífera varieties. Table S6. List of genes identified in the chloroplast genome of *Vitis vinifera*. Table S7. Codon counts per amino acid in the chloroplast genomes of *Vitis vinifera* varieties. Table S8. Codon percentage per amino acid in the chloroplast genomes of *Vitis vinifera* varieties.
