# Supplementary figures and images for: In Silico Genomic Analysis of Chloroplast DNA in Vitis Vinifera L.: Identification of Key Regions for DNA Coding
Source: Genes (Basel). 2025 May 31;16(6):686. doi: 10.3390/genes16060686 (PMC12192835; doi:10.3390/genes16060686)

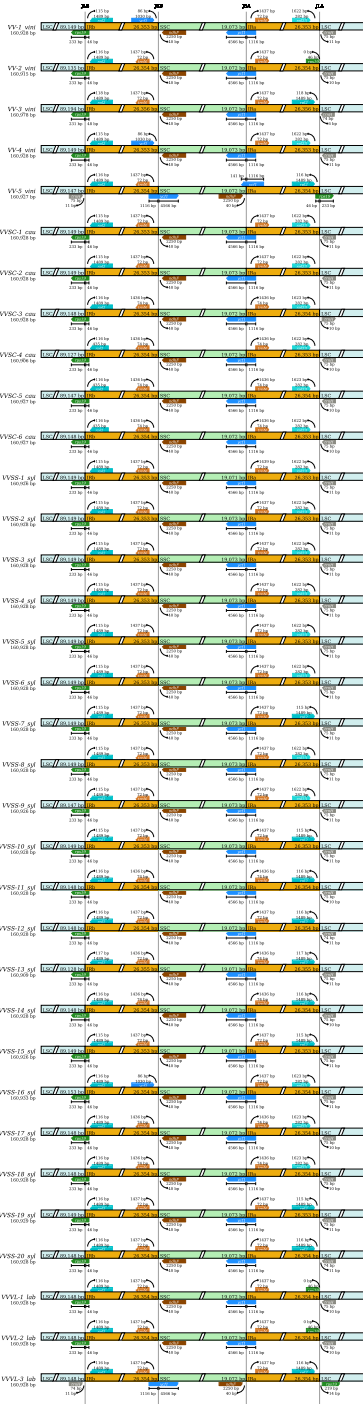

Supplement: Supplementary file 1 [file genes-16-00686-s001.zip › Figure_S1.pdf]

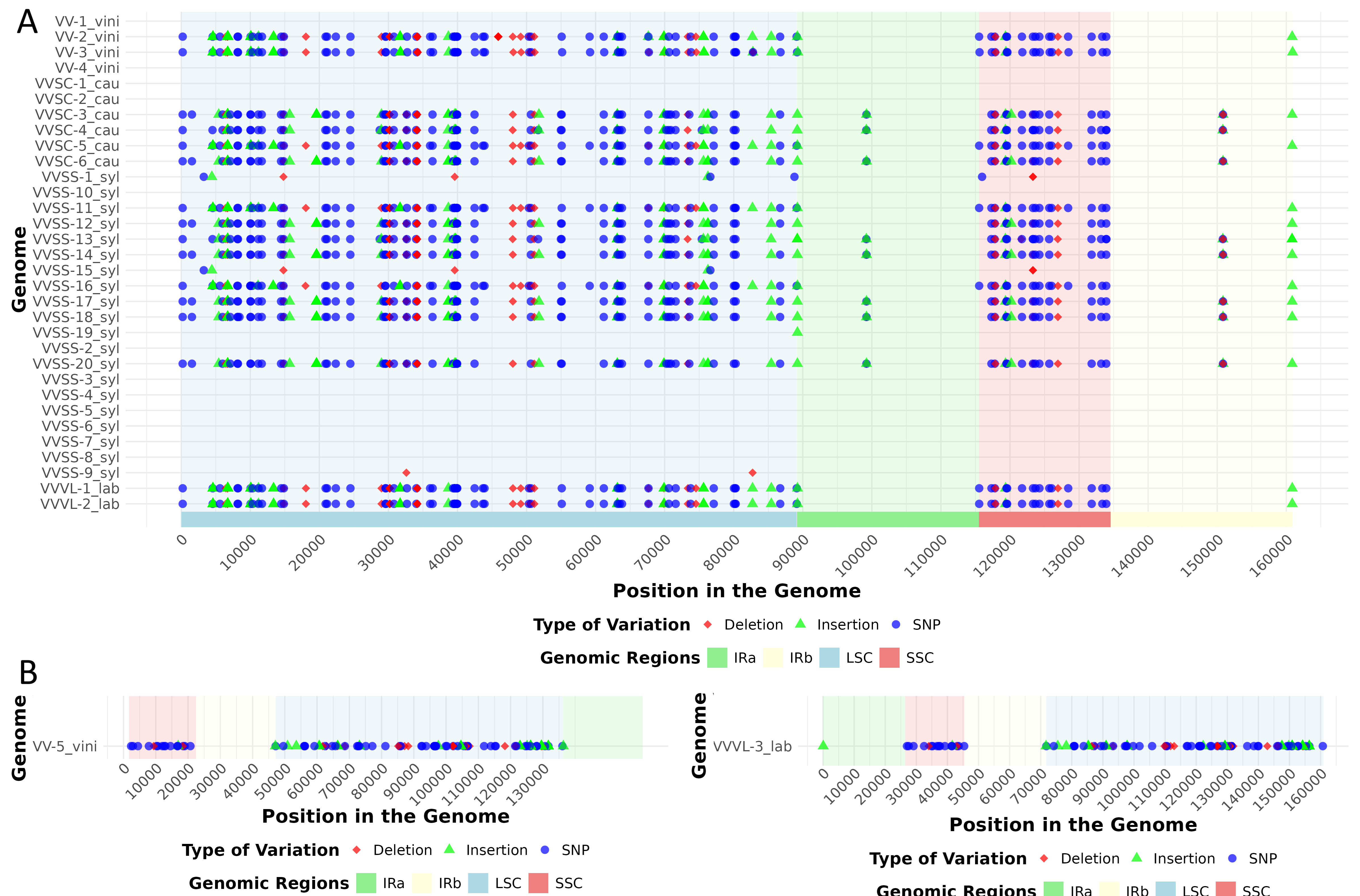

Supplement: Supplementary file 1 [file genes-16-00686-s001.zip › Figure_S2.png]

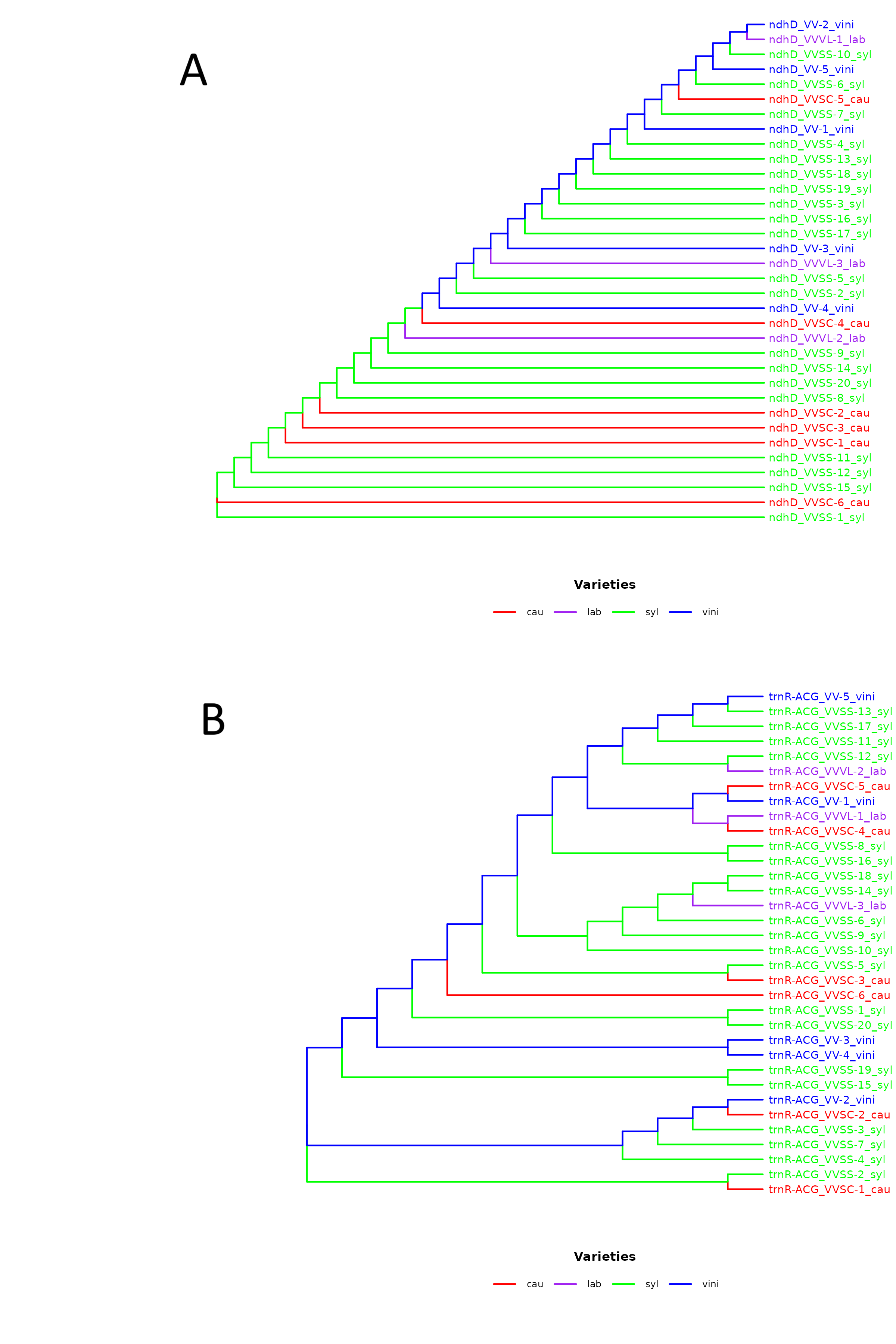

Supplement: Supplementary file 1 [file genes-16-00686-s001.zip › Figure_S3.png]

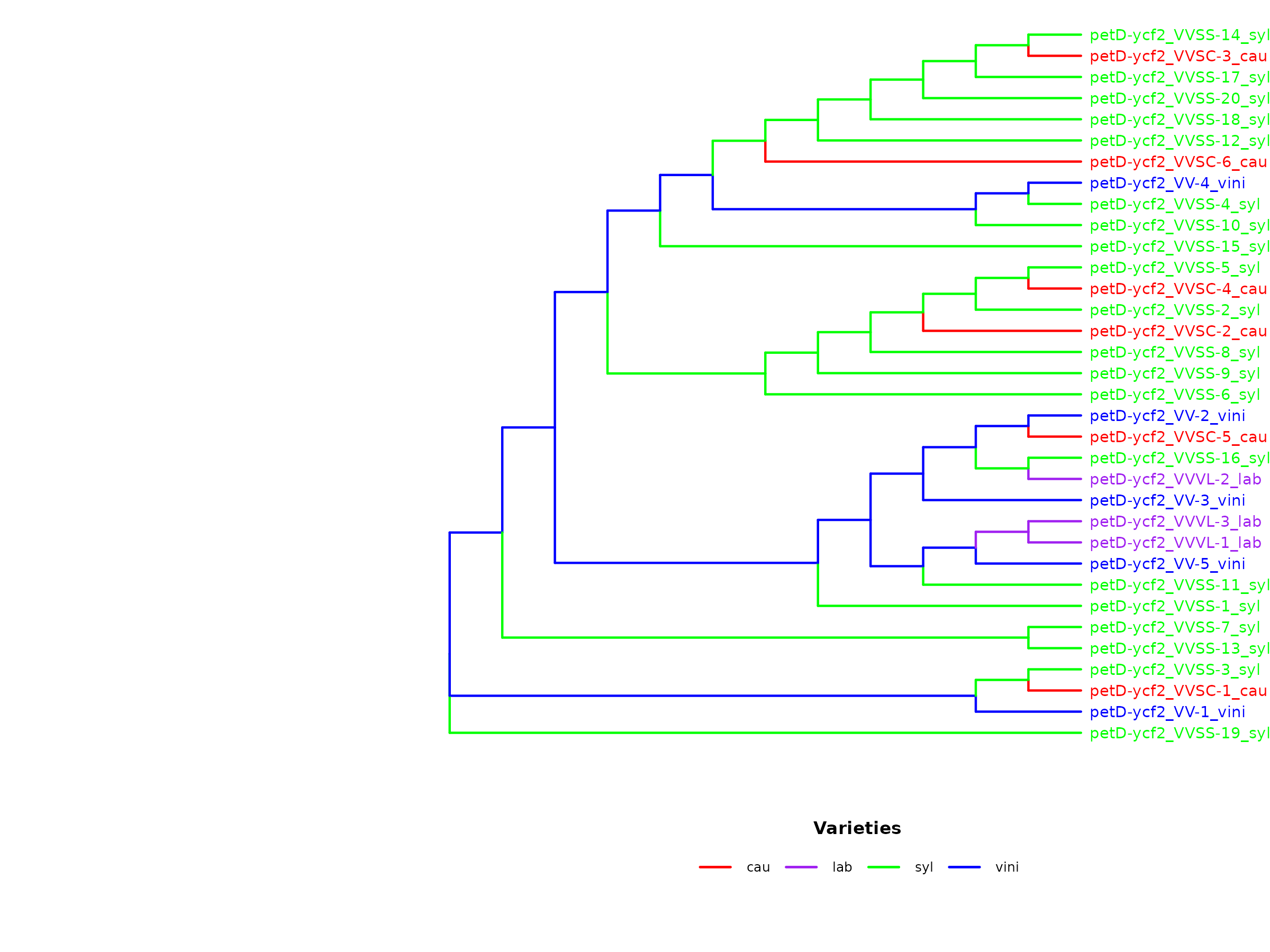

Supplement: Supplementary file 1 [file genes-16-00686-s001.zip › Figure_S4.png]
